# Surface-Based Spontaneous Oscillation in Schizophrenia: A Resting-State Functional Magnetic Resonance Imaging Study

**DOI:** 10.3389/fnhum.2021.750879

**Published:** 2021-12-06

**Authors:** Xianyu Cao, Huan Huang, Bei Zhang, Yuchao Jiang, Hui He, Mingjun Duan, Sisi Jiang, Ying Tan, Dezhong Yao, Chao Li, Cheng Luo

**Affiliations:** ^1^MOE Key Lab for Neuroinformation, High-Field Magnetic Resonance Brain Imaging Key Laboratory of Sichuan Province, The Clinical Hospital of Chengdu Brain Science Institute, University of Electronic Science and Technology of China, Chengdu, China; ^2^High-Field Magnetic Resonance Brain Imaging Key Laboratory of Sichuan Province, Center for Information in Medicine, University of Electronic Science and Technology of China, Chengdu, China; ^3^Research Unit of NeuroInformation (2019RU035), Chinese Academy of Medical Sciences, Chengdu, China; ^4^The Key Laboratory for Computer Systems of State Ethnic Affairs Commission, Southwest Minzu University, Chengdu, China

**Keywords:** schizophrenia, fMRI, spontaneous neuronal activity, surface-base, resting state

## Abstract

Schizophrenia (SZ) is considered as a self-disorder with disordered local synchronous activation. Previous studies have reported widespread dyssynchrony of local activation in patients with SZ, which may be one of the crucial physiological mechanisms of SZ. To further verify this assumption, this work used a surface-based two-dimensional regional homogeneity (2dReHo) approach to compare the local neural synchronous spontaneous oscillation between patients with SZ and healthy controls (HC), instead of the volume-based regional homogeneity approach described in previous study. Ninety-seven SZ patients and 126 HC were recruited to this study, and we found the SZ showed abnormal 2dReHo across the cortical surface. Specifically, at the global level, the SZ patients showed significantly reduced global 2dReHo; at the vertex level, the foci with increased 2dReHo in SZ were located in the default mode network (DMN), frontoparietal network (FPN), and limbic network (LN); however, foci with decreased 2dReHo were located in the somatomotor network (SMN), auditory network (AN), and visual network (VN). Additionally, this work found positive correlations between the 2dReHo of bilateral rectus and illness duration, as well as a significant positive correlation between the 2dReHo of right orbital inferior frontal gyrus (OIFG) with the negative scores of the positive and negative syndrome scale in the SZ patients. Therefore, the 2dReHo could provide some effective features contributed to explore the pathophysiology mechanism of SZ.

## Introduction

Schizophrenia (SZ) is commonly considered as a heterogeneous psychiatric disorder with a wide range of clinical and biological manifestations, which also is included among the world’s top 10 causes of long-term disability (Dong et al., 2017; Luo et al., 2020). These manifestations include positive symptoms (hallucination, delusions, and disturbed emotions), negative symptoms (affective flattening, avolition-apathy, alogia, social withdrawal, and attention impairment), and cognitive dysfunction (processing speed, attention/vigilance, working memory) (Schultz and Andreasen, 1999; Tomasik et al., 2016; Jiang et al., 2019). However, the clear neuronal mechanisms underlying these symptoms remain unclear.

As a number of researchers continue to study SZ, various theories have been proposed to explain these different symptoms. In the study of SZ sensory and perceptual disorders, the bottom-up and top-down brain system integration theory proposes that the bottom-up functional impairment will lead to the abnormality of sensory mechanism (Javitt and Freedman, 2015), whereas the abnormality of top-down cognitive mechanism is the cause of cognitive failure (Allen et al., 2012). Specifically, auditory hallucinations are thought to result from the failure of top-down inhibitory control over bottom-up perceptual processes (Hugdahl, 2009). Furthermore, the pathophysiology underlying SZ has been attributed to functional abnormalities of the brain, which can be partially explained by spontaneous brain activity (Xu et al., 2015; Zhang et al., 2021). Previous studies have demonstrated that the SZ has exhibited functional changes in both task-evoked activation and spontaneous brain activity (Light et al., 2006; Jiang et al., 2020). SZ is regarded as a neurodevelopmental disorder, and its symptoms occur spontaneously. Therefore, investigating spontaneous brain activities in SZ can help us understand the pathophysiology underlying SZ.

The development and application of functional magnetic resonance imaging (fMRI) provide a new way to explore the neural activity of SZ (Biswal et al., 1995; Luo et al., 2020). Several works have revealed abnormal functional connectivity (FC) in patients with SZ, including the default mode network (DMN) (Bluhm et al., 2007; Canuet et al., 2011), the frontoparietal network (FPN) (Chen et al., 2017), thalamus (Wang et al., 2020), hippocampus (Ho et al., 2016), and temporoparietal area (Jiang et al., 2018; Huang et al., 2020); these findings suggested that abnormal neural activity in these region may be the alterations characteristic of SZ. In addition, a review has demonstrated that abnormal spontaneous brain activity in SZ were mainly located in the somatosensory cortex, occipital cortex (OC), medial temporal cortex (MTC), and medial prefrontal cortex (MPFC) (Xu et al., 2015); abnormal activation patterns within these regions may reflect different aspect symptoms in the SZ.

Regional homogeneity (ReHo), which measures the similarity or synchronism of the time series of a given voxel with its neighboring voxel within a single region, is the most widely used to reflect the local neural synchronous spontaneous oscillation in the brain (Zang et al., 2004). Since the blood oxygenation level dependent signal of fMRI reflects neural activity, abnormal ReHo is probably related to the temporal variation of regional spontaneous neural activity (Logothetis and Wandell, 2004). According to previous studies on subjects with a first-episode drug-naïve patients with SZ comorbid with depression (Fang et al., 2021), unipolar depression, and bipolar disorder (Liu et al., 2020) and SZ (Kuhn and Gallinat, 2013), abnormal ReHo may provide new insights into the potential pathophysiological mechanisms of psychiatric disorders. In addition, abnormal neural activities are associated with clinical symptoms and cognitive dysfunction, and ReHo may be used to evaluate the severity of clinical symptoms and cognitive dysfunction (Gao et al., 2020).

In recent years, ReHo has been widely used to investigate SZ pathophysiology (Zalesky et al., 2012; Cui et al., 2016; Zhao et al., 2018). Increased ReHo may represent neural hyperactivity in the brain regional area and vice versa (Zang et al., 2004). However, to our knowledge, all previous studies examined ReHo in patients with SZ to explore the abnormalities of regional functional synchronization at the voxel level (Xu et al., 2015; Chen et al., 2020). This volume-based ReHo (3dReHo) approach ignores the intersubject variability of cortical folding patterns, and voxels near the boundary between the gray and white matter show a significant partial volume effect (Li et al., 2014). A previous work has demonstrated that volume-based smoothing causes contamination of the primary motor cortex by somatosensory cortical responses, leading to false positive motor activation (Brodoehl et al., 2020). Therefore, in our work, we used a surface-based two-dimensional ReHo (2dReHo) method (Bo et al., 2019), which focuses on neural activation in the cerebral cortex, decreases signal contamination considerably between neighboring functional brain regions, and improves the validity of the activity results (Jiang and Zuo, 2016; Brodoehl et al., 2020).

In our work, with this relatively new 2dReHo approach, 97 patients with SZ were recruited to explore the abnormal local functional homogeneity of spontaneous neuronal activity and its correlations with symptomatic severity. We hypothesized that patients with SZ may have extensive abnormal local synchronization of spontaneous neuronal activity across the cortical mantle, and the 2dReHo could provide more accurate and persuasive evidences to expound the pathophysiological hypothesis of SZ.

## Materials and Methods

### Participants

Ninety-seven patients with SZ (29 women, 41.0 ± 11.5 years old) were recruited from the inpatient in the Clinical Hospital of Chengdu Brain Science Institute in University of Electronic Science and Technology of China. The diagnosis of SZ was according to the structured clinical interview for Diagnostic and Statistical Manual of mental disorder (DSM-IV) criteria. All patients were treated with antipsychotics. Clinical symptomatic severity was evaluated by the positive and negative syndrome scale (PANSS) (Kay et al., 1987). In this work, 126 healthy controls (HC) (42 women, 38.0 ± 14.9 years old) who matched in age and gender with SZ were also enrolled from the local community through advertisements. The exclusion criteria for both groups included history of major medical or neurological illness, traumatic brain injury, first- and second-degree relatives with a history of mental illness, current drug or alcohol abuse, and MRI contraindications. Additionally, four SZ patients and one HC were excluded because they failed to accomplish all of T1 and resting-state fMRI data acquisitions, or their head-motion was beyond 2 mm or 2°. These patients were part of our previous studies and more details can be found in prior published works (Jiang et al., 2020). Written informed consents were signed by all participants before the MRI scanning, and the Ethics Committee of the Clinical Hospital of Chengdu Brain Science Institute approved the work (No. CDFH2014030501).

### Data Acquisition

High-resolution T1-weighted images and Resting-state fMRI were collected on a 3.0 Tesla scanner (GE Discovery MR 750) with an 8-channel standard whole head coil at the MRI Center of University of Electronic Science and Technology of China. During the scanning, sponges were used to reduce the noise of head movements, and participants were requested to keep their minds wandering during the resting-state scan with eyes closed without falling asleep. Three-dimensional T1-weighted data were obtained by using a fast-spoiled gradient echo sequence [repetition time (TR) = 6.008 ms; echo time (TE) = 1.984 ms; flip angle (FA) = 9°; field of view (FOV) = 25.6 × 25.6 cm^2^; matrix size = 256 × 256; slice thickness = 1 mm, no gap, and slice number = 156]. Resting-state functional data were obtained with the use of a gradient-echo echo-planar imaging (EPI) sequence (TR = 2,000 ms; TE = 30 ms; FA = 90°; FOV = 24 × 24 cm^2^; matrix size = 64 × 64; slice thickness = 4 mm, no gap and slice number = 35). Scanning time lasting 510 s (255 volumes).

### Data Preprocessing

Both the structural and functional image preprocessing were performed using a surface-based resting-state fMRI data analysis toolbox (DPABISurf^1^, Yan et al., 2016). The structural image processing steps consisted of: (1) spatial adaptive non-local mean filter was used to remove MRI spatial noise (Xing et al., 2011), (2) intensity correction, (3) skull-stripped, (4) brain tissue segmentation, cerebrospinal fluid (CSF), white matter (WM), and gray matter (GM), (5) brain surface reconstruction *via* the recon-all command in FREESURFER (version 6.0.1) (Dale et al., 1999), and (6) spatial normalization from individual native space to fsaverage space (Fischl, 2012). The functional image processing steps consisted of: (1) remove the first 10 volumes, (2) skull-stripped, (3) slice timing correction, (4) head motion correction, (5) nuisance correction by regressing out the WM and CSF mean time series according the WM and CSF masks segmented by FREESURFER as well as the Friston-24 motion time series (Friston et al., 1996), except for the global signal, as it may be the basis for alterations in neural information flow in SZ patients (Yang et al., 2014), (6) band-pass temporal filtering (0.01–0.1 Hz), (7) boundary-based registration (BBR) algorithm was used to register individual structural and functional images (Greve and Fischl, 2009), and (8) projection of the individual preprocessed volume-based function image onto a standard cortical surface (fsaverage5). Specifically, we calculated the midpoints of each pair of vertices on the pia meningeal and white matter surfaces generated by FREESURFER. Through BBR algorithm, we could get the registration matrix between the native fMRI volume and structural volume of each subject. Then, we choose fsaverage5 surface space as the target surface for projection and interpolation. For each subject’s vertex in the fsaverage5 space, its corresponding coordinates in the native structural space were calculated first, and then its corresponding coordinates in the fMRI space were calculated. Finally, according to these given coordinates, the fMRI values are interpolated using the trilinear interpolation method.

### Cortical Surface-Based 2dReHo

In our study, 2dReHo was used to explore the abnormal local functional homogeneity of spontaneous neuronal activity. For each subject, the 2dReHo is obtained by calculating Kendall’s concordance coefficient (KCC) of the given vertex and the nearest neighbors’ time series (Zuo et al., 2013). This calculation was repeated for all vertices on both hemispheres, resulting in a 2dReHo map of both hemispheres. Specifically, for each vertex, we determined its 18 nearest vertices, based on which we calculated the KCC of fMRI time series of all 19 vertices. Of note, if we try to keep the same length of neighbors for certain vertices or voxel, the number of neighbors is different; for 2dReHo, the length-one and length-two calculation recruits 6 and 18 neighbors, respectively, whereas the 3dReHo recruits 26 neighbors. Moreover, previous studies have shown that the spatial pattern of length-two neighbors 2dReHo is highly similar to length-one neighbors 2dReHo’s pattern (Zuo et al., 2013). Based on the above reasons, we chose 18 vertices as our calculation criteria. Finally, Gaussian spatial smoothing was performed for the 2dReHo maps using a 10-mm full-width half maximum Gaussian kernel (Zuo et al., 2013).

### Statistical Analysis

The demographic and psychometric data were analyzed using SPSS 28.0. Two-sample *t*-tests were used to compare age and years of education. The chi-square test was performed to compare the difference in the gender between SZ and HC groups.

To compare the 2dReHo differences between the SZ and HC groups, the two-sample *t*-test was performed between the surface maps of SZ and HC groups by DPABISurf. Multiple comparisons correction was performed using the false discovery rate (FDR) corrected. The 2dReHo in the regions with significant differences between the two groups were extracted. Subsequently, the Spearman rank correlation analysis was used to evaluate the relationship between the 2dReHo in these regions and clinical variables (illness duration, PANSS subscales, and total scores) after regressing out age and gender.

## Results

### Demographic and Clinical Characteristics

The remaining 93 patients with SZ (28 women, 40.0 ± 11.5 years old) and 125 controls (41 women, 37.6 ± 14.9 years old) were matched in age (*p* = 0.09), gender (*p* = 0.67), and education years (*p* = 0.08). More demographic and clinical information of the patients are summarized, as shown in Table 1.

**TABLE 1:** Demographic data for SZ vs. HC participants.

**Characteristic**	**Patients**		**Health controls**		***P*-value**
	**Mean**	**SD**	**Mean**	**SD**	
Number	97		126		
Gender (male/female)	68/29		84/42		0.67^a^
Age (years)	41.0	11.5	38.0	14.9	0.10^b^
Education (years)^c^	11.8	3.1	10.9	3.4	0.08^b^
Duration of illness (years)	16.3	10.9			
Chlorpromazine equivalents (mg/day)	324.5	157.1			
PANSS score					
PANSS-positive^d^	13.32	5.89			
PANSS-negative^d^	20.70	6.06			
PANSS-general^d^	28.19	5.86			
PANSS-total^d^	62.21	13.26			

*PANSS, positive and negative syndrome scale; SZ, schizophrenia; HC, healthy controls.*

*^*a*^χ^2^ test.*

*^*b*^Two-sample *t*-test.*

*^*c*^Data of 76 patients and 111 controls available.*

*^*d*^Data of 64 patients available.*

### Surface-Based 2dReHo Differences Between Schizophrenia and Healthy Controls

**FIGURE 1:**
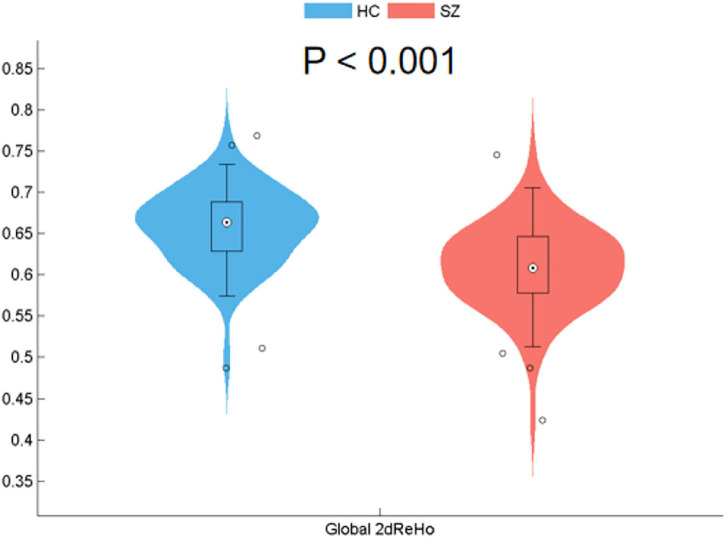
Reduced surface-based 2dReHo in SZ patients compared with HC at the global level (*p* < 0.001) across the cortical surface. Global 2dReHo, global mean two-dimensional regional homogeneity. SZ, schizophrenia; HC, healthy control.

At the global level, the patients with SZ showed a significant reduction in the global 2dReHo of the cortical surface (*t* = −7.336, *p* < 0.001) compared with HC (Figure 1).

**FIGURE 2:**
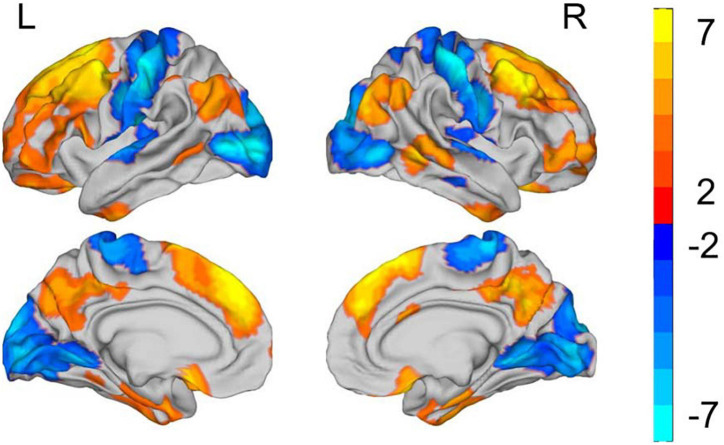
Group differences in the surface-based 2dReHo. L refers to **left side** of brain. R refers to **right side** of brain. All results are shown after FDR corrected (*p* < 0.05). The color bar represents T values of the two-sample *t*-test; the yellow regions represent higher and blue regions represent lower 2dReHo in the patient group compared with the control group.

At the vertex level, patients with SZ showed significantly increased 2dReHo in regions widely distributed across DMN (bilateral middle temporal gyrus and precuneus), FPN (bilateral frontal and parietal cortex), and limbic network (LN) (inferior temporal gyrus and rectus) (Figure 2 and Table 2). Moreover, reduced 2dReHo was observed in the somatomotor network (SMN) (bilateral postcentral gyrus), auditory network (AN) (heschl and superior temporal gyrus), and visual network (VN) (bilateral occipital cortex) in SZ group (Figure 2 and Table 2).

**TABLE 2:** Group differences in the surface-based 2dReHo.

**Brain regions (peak index) (AAL)**	**Peak index^a^**	**Peak T value**	**Cluster size (mm^2^)^b^**
**Left hemisphere**			

Default mode network			
Frontal_Mid_L	8,062	6.95	8154.28
Precuneus_L	9,718	4.49	1607.18
Angular_L	5,108	4.23	1116.1
Temporal_Mid_L	4,344	3.18	138.16
Frontoparietal network			
Parietal_Inf_L	2,044	4.16	106.04
Limbic network			
Rectus_L	430	5.11	925.2
Temporal_Inf_L	5,539	3.76	846.3
Somatomotor network			
Postcentral_L	8,726	–7.02	5664.69
Visual network			
Occipital_Mid_L	5,283	–7.59	5904.62
Auditory network			
Temporal_Sup_L	1,172	–5.66	730.33

**Right hemisphere**			

Default mode network			
Frontal_Mid_R	3,607	6.35	4417.62
Precuneus_R	9,354	5.15	1561.81
Angular _R	784	4.39	1298.13
Temporal_Mid_R	5,640	4.45	736.1
Frontoparietal network			
Frontal_Inf_Orb_R	7,613	4.02	1130.25
Limbic network			
Rectus_R	514	4.96	813.26
Temporal_Inf_R	5,486	3.97	657.19
Somatomotor network			
Postcentral_R	1,071	–7.54	5387.76
Visual network			
Occipital_Sup_R	6,872	–6.37	6116.6
Auditory network			
Heschl_R	1,309	–3.28	260.62

*L, left side of brain; R, right side of brain; AAL, automated anatomical labeling atlas.*

*^*a*^The index of the vertex with the peak T value.*

*^*b*^The cluster size represents the number of vertices within the cluster.*

### Relationships Between Surface-Based 2dReHo and Clinical Variables

**FIGURE 3:**
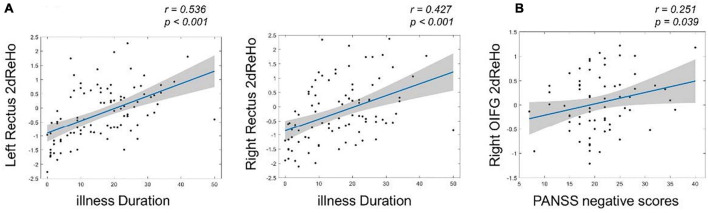
The relationships between surface-based 2dReHo and clinical variables in patients with SZ by using Spearman rank correlation analysis. **(A)** The left and right rectus 2dReHo were significantly positive correlated with the illness duration (FDR corrected, *p* < 0.05); **(B)** the right OIFG 2dReHo exhibited significant positive correlation with the PANSS negative scores. OIFG, orbital inferior frontal gyrus.

After multiple comparisons correction by the FDR (*p* < 0.05, FDR corrected), the 2dReHo of left rectus (*r* = 0.536, *p* < 0.001) and right rectus (*r* = 0.427, *p* < 0.001) showed significantly positive correlations with illness duration in the SZ group. Moreover, the 2dReHo in the right orbital inferior frontal gyrus (OIFG) (*r* = 0.251, *p* = 0.039, without corrected) exhibited significant positive correlation with the PANSS negative scores (Figure 3).

## Discussion

To the best of our knowledge, this is the first work to investigate the abnormal local functional homogeneity of spontaneous neural oscillation in patients with SZ. At the global level, our work indicated that compared with HC, the SZ patients showed significantly reduced global 2dReHo. As for the vertex level, we demonstrated significantly enhanced 2dReHo in the DMN, FPN, and LN, and also significantly reduced 2dReHo in the primary perception networks (SMN, AN, and VN) in SZ. These results suggested that the SZ is associated with abnormal synchronization of spontaneous neural oscillation in the regional brain. Moreover, the relationships between the 2dReHo abnormalities and clinical variables, including illness duration, PANSS subscales, and total scores were evaluated in patients with SZ. The results indicated that the 2dReHo of bilateral rectus were significantly positive related with illness duration, and also the 2dReHo in the right OIFG exhibited significant positive correlation with the PANSS positive scores.

Recently, several studies have investigated the ReHo abnormalities in SZ, but with inconsistent results. The reasons for these inconsistencies are complex and it is necessary to reconcile the heterogeneity of different findings. Of note, our results are highly consistent with those of a previous metaanalysis on 3dReHo in the SZ (Xu et al., 2015), which demonstrated an increased spontaneous brain activity in the striatum, MTC, and MPFC, and a decreased activity in the sensorimotor and visual cortex. The results indicated that our 2dReHo results may provide more accurate and persuasive evidences to expound the abnormal synchronization of spontaneous neural activity of SZ. Moreover, at the vertex level, the patients with SZ exhibited decreased 2dReHo in the SMN, the precentral gyrus, and the postcentral gyrus, which was consistent with previous studies (Liu et al., 2006). This finding was understandable as patients with SZ commonly illustrate a variety of symptoms, including psychomotor and fine motor, and also abnormalities in touch, temperature, pain, tension, and vibration (Chen et al., 2015). In addition, the posterior central gyrus may be associated with the processing of multimotion-related cognitive functions, which was abnormal in SZ (Elvevag and Goldberg, 2000). In general, patients with SZ exhibited reduced global 2dReHo across the cortical surface, which revealed that SZ showed deactivation in synchronization of spontaneous neural oscillation across the global cortices.

Patients with SZ exhibited reduced 2dReHo in the occipital lobe, which may serve as the underlying functional basis of visual preliminary processing defects, such as perceptual closure, object recognition, and face processing (Pastrnak et al., 2017). The same reduction exists in drug-naive patients with bipolar disorder (Fang et al., 2021). Notably, OC is the main region of extrastriate body area (EBA), and the functional abnormalities of OC may lead to error, which selectively responds to the viewing of body parts and mental imagery of embodied self-location. Previous analysis showed that the brain injury of EBA was mainly related to the autoscopic phenomena (Heydrich and Blanke, 2013). Therefore, the relative reduction of 2dReHo in the OC may impair the perception of body ownership in patients with SZ.

Interestingly, a significant increase of 2dReHo was found in widespread brain regions, including the bilateral frontal and parietal, temporal cortex, and precuneus. Actually, increased ReHo in the medial frontal cortex (MFC) in SZ seems to contradict some previous fMRI findings (Hill et al., 2004; Whalley et al., 2008; Pomarol-Clotet et al., 2010); however, consistent with a metaanalysis of depressive patients, increased ReHo in the superior and inferior frontal gyrus were associated with depressive symptoms (Iwabuchi et al., 2015; Fang et al., 2021). This might be explained by the hypotheses that 2dReHo can obtain more neglected information about local activation of the brain compared with ReHo (Li et al., 2014; Jiang and Zuo, 2016). In addition, the reliability of hemodynamic-induced prefrontal dysfunction in SZ seems controversial. A recent study also showed differences in ReHo among different types of SZ (Gao et al., 2018). Furthermore, several current studies have reported increased activation in the prefrontal cortex in SZ responding to performing working memory tasks (Tan et al., 2006, 2007). In fact, the MFC in patients with SZ shows task-related inactivation failure (Whitfield-Gabrieli et al., 2009). Our finding of increased 2dReHo in MFC may explain the hyperfrontality responding to abnormality of task-performance in the patients of SZ. Moreover, a previous study of electroconvulsive therapy (ECT) in depressive patients also showed a decrease in ReHo values in bilateral SFG after ECT.

Our study demonstrated widespread abnormalities in the consistent activation of local brain regions in patients with SZ, including two higher-order intrinsic brain networks (DMN, FPN), and some low-level networks (SMN, VN). The DMN and FPN are two higher-order intrinsic brain networks with functional heterogeneities, which appear to be crucial for both daily general functioning and physiopathology (Khadka et al., 2013). DMN, the most prominent network at rest, can be considered as the baseline of brain processing (Calhoun et al., 2009; Whitfield-Gabrieli and Ford, 2012). The DMN often shows deactivation during tasks requiring external attention, while it increases activity during unconstrained thought (Mason et al., 2007), introspection (Svoboda et al., 2006), and self-related processing (Lin et al., 2011). Failure of this function may result in individuals mistakenly attributing internally generated thoughts as exogenous (Frith, 1995). For FPN, it is often evoked by various cognitive tasks (Dosenbach et al., 2007; Cole et al., 2013), and multiple executive functions subserved by the FPN including working memory and sustained attention (Das et al., 2020). The dysfunction of FPN may be one of the abnormal neural mechanisms of SZ (Dong et al., 2018, 2019). In addition, this work found that the 2dReHo of right OIFG in FPN was also significantly positively correlated with the PANSS negative scores, and bilateral rectus was associated with illness duration, which provided evidence that the local brain activity might predict the severity of SZ. Furthermore, a previous work showed the disconnection between DMN and FPN in SZ. Specifically, the connectivity between the FPN and the DMN decreased with greater working memory load in healthy participants, but increased in patients with SZ (Godwin et al., 2017). This was consistent with our results that both FPN and DMN were hyperactivated in SZ. Moreover, the decrease of 2dReHo in the low-level primary perceptual networks including SMN, AN, and VN indicates that the consistency of spontaneous activation is reduced, which were well-documented for the deficits of perceptual processing and multisensory integration in SZ.

It would be noted that the resting state fMRI features such as ReHo, which reflect the regional neural spontaneous oscillation in temporal and/or spatial domain, the most widely used to investigate the potential pathophysiological mechanisms of various psychiatric disorders (Zang et al., 2004). However, many of the studies in these illnesses reported similar alteration of neural spontaneous oscillation in DMN and FPN (Zuo et al., 2013; Jiang and Zuo, 2016), which would reflect that the domain-general function was interrupted due to common and/or similar symptoms across psychiatric disorders. Thus, domain-specific network or its relationship with domain-general function might be important to the specific psychiatric disorder. In general, the disrupted interaction between primary perception system and high-order cognitive function was found in SZ rather than depression or bipolar disorder (Dong et al., 2018). Consistent with the hypotheses of uncoupling between high- and low-order brain functional networks in SZ, the opposite alteration between high-order networks (increased in DMN and FPN) and low-order networks (decreased in VN, AN, and SMN) was observed in this work, which might provide a specific insight to understand the potential pathophysiological mechanisms of SZ.

There are several limitations in this study. First, all patients were medicated and the negative symptoms were dominant, which may lead to a certain bias in our results. Second, our present 2dReHo approach can only analyze functional abnormalities in the cortex rather subcortical regions, but the subcortical regions have been proven to be key regions in the emotional regulation circuit. Third, the instructions associated with the resting state approach may have potential impact, leading to confounding results. Fourth, the physiological origin and functional significance of ReHo remain unclear, which limits the exploration of the physiological basis of 2dReHo.

## Conclusion

Using the 2dReHo method, SZ patients showed enhanced surface-based spontaneous neural oscillation in two higher-order functional networks including DMN and FPN, as well as reduced 2dReHo in the low-order perceptual networks: SMN, AN, and VN. Dysfunction of these functional networks was closely related to the symptoms of SZ. Therefore, these findings indicated that the 2dReHo might provide the effective approach to explore the pathophysiology mechanism of SZ.

## Data Availability Statement

The raw data supporting the conclusions of this article will be made available by the authors, without undue reservation.

## Ethics Statement

The studies involving human participants were reviewed and approved by the Ethics Committee of the Clinical Hospital of Chengdu Brain Science Institute. The patients/participants provided their written informed consent to participate in this study. Written informed consent was obtained from the individual(s) for the publication of any potentially identifiable images or data included in this article.

## Author Contributions

YT, DY, and CLu designed the study and supervised the project. CLi, XC, and MD managed the experiments and data collection. YJ, HHe, BZ, HHu, and XC undertook the data analysis. XC, HHu, YT, YJ, and CLu wrote and revised the manuscript. All the authors reviewed the manuscript and approved the final manuscript.

## Conflict of Interest

The authors declare that the research was conducted in the absence of any commercial or financial relationships that could be construed as a potential conflict of interest.

## Publisher’s Note

All claims expressed in this article are solely those of the authors and do not necessarily represent those of their affiliated organizations, or those of the publisher, the editors and the reviewers. Any product that may be evaluated in this article, or claim that may be made by its manufacturer, is not guaranteed or endorsed by the publisher.

## Footnotes

1
http://rfmri.org/DPABISurf
